# Real-world efficacy and safety of PD-1 inhibitors in patients living with HIV and cancer: a retrospective cohort study

**DOI:** 10.3389/fonc.2026.1846602

**Published:** 2026-06-24

**Authors:** Xiaola Xue, Juyi Wu, Zhenpeng Tan, Chunyu Tian, Qiong Li, Shujuan Zhou, Yuchao Xia, Shaojie Yang, Xuan Yang

**Affiliations:** 1 Department of Pharmacy, Henan Infectious Diseases Hospital, the Sixth People’s Hospital of Zhengzhou, Zhengzhou, China; 2 Department of Oncology, Henan Infectious Diseases Hospital, the Sixth People’s Hospital of Zhengzhou, Zhengzhou, China; 3 Department of Clinical Research Center, Henan Infectious Diseases Hospital, the Sixth People’s Hospital of Zhengzhou, Zhengzhou, China; 4 Department of Infectious Disease, Henan Infectious Diseases Hospital, the Sixth People’s Hospital of Zhengzhou, Zhengzhou, China

**Keywords:** advanced cancer, efficacy, immunotherapy, people living with HIV, programmed cell death 1 receptor, treatment-related adverse events

## Abstract

**Objective:**

Programmed cell death protein 1 (PD-1) inhibitors have established a cornerstone in cancer therapy; however, there remains a lack of data regarding their use in people living with human immunodeficiency virus (HIV) infection (PWH), especially in patients with low CD4^+^ T-cell counts or baseline detectable HIV RNA. This study assessed the real-world efficacy and safety of PD-1 inhibitors in PWH presenting with concurrent malignancies, specifically capturing individuals with advanced baseline immunosuppression (CD4^+^ T-cell count < 100 cells/μL, *n* = 4) or with detectable HIV RNA (*n* = 9).

**Methods:**

From January 2022 to December 2024, patients diagnosed with cancer and treated with PD-1 inhibitors (camrelizumab, sintilimab, or tislelizumab) were enrolled. The included population was divided into an HIV-positive group and an HIV-negative group based on HIV status. Demographic details, clinical data, and cancer status were collected. Using propensity score matching (PSM) to adjust for baseline imbalances, we compared median progression-free survival (PFS), objective response rate (ORR), disease control rate (DCR), and treatment-related adverse events (TRAEs) between the two groups. Cox regression analysis was conducted to evaluate the influence of multiple variables on treatment response.

**Results:**

From 90 enrolled patients (70 HIV-positive, 20 HIV-negative), PSM selected 56 (37 HIV-positive, 19 HIV-negative) for final analysis. The ORR and DCR were comparable between the HIV-positive and HIV-negative groups (16.2% vs. 15.8% and 51.4% vs. 47.4%, respectively). Median PFS was 11.5 months (95% CI: 6.8–16.2) for the HIV-positive group and 16.9 months (95% CI: Not evaluated (NE)–40.4) for the HIV-negative group. For the lung cancer subgroup, the ORR and DCR were 23.5% and 70.6% among 17 HIV-positive patients vs. 30.0% and 50.0% among 10 HIV-negative patients. The respective median PFS was 14.5 months (95% CI: 4.9–24.2) and 17.2 months (95% CI: NE–36.0). Multivariable Cox regression analysis confirmed that HIV status was not independently associated with PFS in the overall cohort. Among the four patients with low baseline CD4^+^ T-cell counts, the DCR was 25.0% (one stable disease [SD], three progressive disease [PD]). Among those with detectable baseline HIV RNA (*n* = 9), four achieved SD and five experienced PD, yielding no objective responses. In the matched cohort, TRAEs were predominantly grades 1–2; severe (grade ≥ 3) toxicities occurred in three of 56 patients (5.4%), all within the HIV-positive group. Four patients—all from the HIV-positive group—discontinued immunotherapy due to TRAEs. HIV virological control was maintained without rebound throughout the study; overall CD4^+^ T-cell counts remained largely stable, with all patients presenting with baseline advanced immunosuppression (CD4^+^ T-cell count < 100 cells/μL) demonstrating robust immune restoration by week 24.

**Conclusion:**

This retrospective study indicated that PD-1 inhibitors are effective and generally well tolerated in PWH with concurrent malignancies. HIV status was not an independent prognostic factor for treatment outcomes, and robust immune restoration was achievable even in patients with advanced baseline immunosuppression. Although severe (grade ≥ 3) TRAEs and treatment discontinuations occurred exclusively within the HIV-positive cohort, overall toxicity rates remained low. Larger prospective studies are required to further validate these findings in patients with low CD4^+^ T-cell counts or unsuppressed viral loads.

## Introduction

By 2024, approximately 40 million people living with human immunodeficiency virus (HIV) infection (PWH) were documented globally. With the expanding accessibility of combination antiretroviral therapy, this population now has a substantially prolonged life expectancy, with more than one in five individuals projected to be over the age of 65 by 2030 (1, 2). Consequent to this increased longevity, the burden of noninfectious chronic diseases, particularly malignant neoplasms, has become progressively severe (3, 4). Research has shown that PWH experience a disproportionately high prevalence of various malignancies. Specifically, the incidence of lung cancer is three- to fivefold higher than that observed in the general population, whereas the risk of non-Hodgkin lymphoma and liver cancer is elevated 8.4- and 5.6-fold, respectively (5, 6). Furthermore, PWH exhibit similarly heightened incidence rates for breast, colorectal, and prostate cancers (7–9). Compounding these challenges, HIV-positive individuals face a markedly elevated risk of cancer-related mortality (10), making malignancies a leading cause of death in this population (11).

The treatment regimens for PWH are similar to those for HIV-negative individuals (12), encompassing surgery, chemotherapy, targeted therapy, and immunotherapy. The advent of immune checkpoint blockade, particularly PD-1 inhibitors, has revolutionized cancer therapeutic strategies and demonstrated great promise across a variety of malignancies (13–16). PD-1 is a cell surface receptor that acts as a T-cell checkpoint and plays a central role in regulating T-cell exhaustion. Blocking the binding of PD-1 to its ligand effectively restores the effector function of specific CD4^+^ and CD8^+^ T cells during the early chronic phase of infection (17). Notably, PD-1 expression also contributes to the promotion of latent HIV infection and favors the maintenance of the HIV reservoir (18). In cancer patients, the binding of PD-1 to its ligand PD-L1 facilitates tumor immune evasion by allowing neoplastic cells to escape immune surveillance (19). Theoretically, PWH with cancer may derive greater therapeutic benefit from PD-1 inhibitors due to this dual therapeutic mechanism. However, concerns regarding potential impaired efficacy in immunodeficient individuals, the aggravation of immune reconstitution inflammatory syndrome in PWH who have recently initiated Antiretroviral therapy (ART), unknown effects on HIV-related opportunistic infections, and potential drug–drug interactions with concurrent ART have historically led to the systematic exclusion of PWH from pivotal clinical trials (20). Although these concerns were clinically grounded, their unintended consequence has been a near-complete absence of high-level evidence for PD-1 inhibitors in the HIV-positive cancer population. This evidence gap creates substantial uncertainty in risk–benefit assessments, leaving clinicians without formal guidance when considering immunotherapy for PWH with cancer.

Several studies have since been conducted to evaluate PD-1 inhibitors in PWH with cancer (21–23), and a recent systematic review synthesized the emerging evidence that immune checkpoint inhibitors exhibit acceptable safety profiles and encouraging antitumor activity in this population (24). Nevertheless, historical inclusion criteria have remained highly restrictive. For example, patients with CD4^+^ T-cell counts below 100 cells/μL were routinely excluded, as were those with detectable HIV RNA. These restrictions, although intended to minimize safety risks, have systematically precluded the very subgroups that face the highest cancer burden and harbor the greatest unmet therapeutic need. Patients with severe lymphopenia are at increased risk of aggressive malignancies and have limited treatment options, yet they remain virtually absent from the current evidence base. Similarly, individuals with uncontrolled HIV replication are frequently excluded, despite the theoretical possibility that PD-1 blockade could restore compromised antiviral immunity. Furthermore, existing studies remain constrained by small sample sizes, limiting the generalizability of their findings.

To address these critical evidence gaps, we conducted a retrospective study utilizing expanded, highly inclusive eligibility criteria—enrolling PWH irrespective of baseline CD4^+^ T-cell counts or HIV viral loads—with a relatively large sample size. Our aim was to provide real-world data on the safety and efficacy of PD-1 inhibitors in PWH with cancer, with particular emphasis on traditionally excluded subgroups—specifically individuals with profound lymphopenia or detectable HIV RNA replication—to directly inform clinical decision-making and facilitate hypothesis generation for future prospective trials.

## Methods

### Study design and patient population

This retrospective, population-based cohort study utilized data from Henan Provincial Infectious Disease Hospital. Henan Province, one of China’s most densely populated regions, bears a heavy HIV/AIDS burden stemming from historical transmission clusters. The hospital serves as a provincial-level clinical research and treatment center specializing in the management of HIV-infected individuals. The study was approved by the Institutional Ethics Committee of Henan Provincial Infectious Disease Hospital (Approval No. IEC-KY-2025-34-01).

This study included patients who presented with cancer and received standardized treatment with PD-1 inhibitors (sintilimab, tislelizumab, and camrelizumab) between January 2022 and December 2024. All PD-1 inhibitors were administered intravenously at standard doses per Chinese clinical guidelines: camrelizumab at 200 mg, sintilimab at 200 mg, or tislelizumab at 200 mg, each administered every 3 weeks. The choice of a specific PD-1 inhibitor was based on the physician’s preference, drug availability, and insurance coverage. Inclusion criteria included the following: (a) confirmed HIV infection; (b) pathological or cytological diagnosis of malignancy; (c) received at least two cycles of PD-1 inhibitors (camrelizumab, sintilimab, or tislelizumab); and (d) available baseline and follow-up data for efficacy and safety assessment. The exclusion criteria were as follows: (a) patients with uncontrolled opportunistic infections or autoimmune disease; (b) receiving PD-1 inhibitors only once; (c) irregular use of PD-1 inhibitors; (d) loss to follow-up; (e) receiving PD-1 inhibitors as monotherapy. To balance baseline clinical characteristics, propensity score matching (PSM) was performed using a variable matching ratio of up to three cases per one control (maximum 3:1 ratio) with a caliper width of 0.2. The matching algorithm accounted for the following baseline variables: sex, age, tumor type, cancer stage, PD-1 inhibitor, treatment line, and number of treatment cycles.

**Figure 1:**
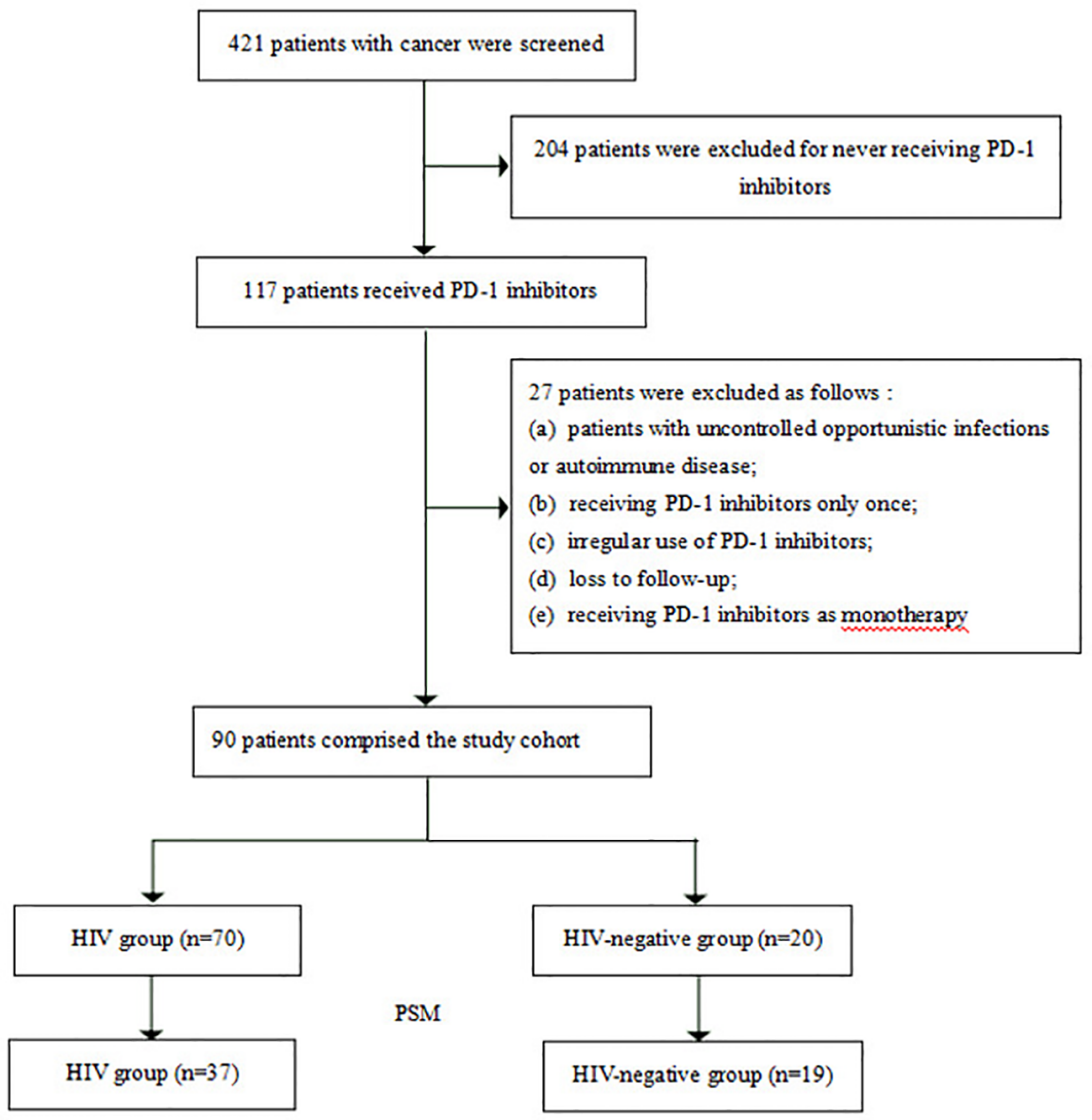
Flow diagram of patient screening and inclusion.

Patients received PD-1 inhibitors as first-line therapy if they had no prior systemic anticancer treatment for advanced/metastatic disease and as second-line or later therapy if they had progressed on prior chemotherapy/targeted therapy. The decision was made by a multidisciplinary tumor board in accordance with National Comprehensive Cancer Network (NCCN) or Chinese Society of Clinical Oncology guidelines. A flowchart summarizing the patient selection process is presented in **Figure 1**.

### Treatment

All patients were treated with PD-1 inhibitors every three weeks according to the standard guidelines and drug instructions, until disease progression, unacceptable toxicity, or patient withdrawal. Patients were regularly followed up and assessed for clinical response. Tumor response was evaluated using RECIST 1.1 for solid tumors and the refined Lugano classification for lymphoma (25, 26). Outcomes were categorized as complete response, partial response, stable disease, or progressive disease, per established response definitions. The cut-off date for follow-up was December 2025.

Safety was assessed using Common Terminology Criteria for Adverse Events (CTCAE v.4.0) (27). Data were collected before and after PD-1 inhibitor treatment every 3 weeks based on medical record review. Data available on the case report form were extracted from electronic health records in the hospital information system. These include characteristics (sex, age, cancer types and stages, previous antitumor regimen), as well as immunotherapy and the number of cycles received. For the HIV-positive group, data on antiretroviral therapy, baseline CD4^+^ T-cell counts, and HIV viral load were also included.

### Statistical analyses

SPSS (version 27) was used for statistical analysis of demographic data, including mean, standard deviation, and percentage. Categorical variables were compared using the Chi-square test or Fisher’s exact test, as appropriate. The Kaplan–Meier method was used for survival analysis. Progression-free survival (PFS) was calculated from the time of initiation of anti-PD-1 antibody therapy until the date of progression, death, or censoring, as applicable. Univariable and multivariable Cox regression analyses were conducted to evaluate the influence of multiple variables on treatment response.

Regarding HIV RNA measurements, 13 of 70 PWH had missing baseline viral load data due to a lack of routine testing before PD−1 inhibitor initiation; these cases were excluded from the subgroup analysis of HIV RNA monitoring but included in the main cohort analysis. For CD4^+^ T-cell counts, one patient had missing data and was therefore excluded from CD4^+^ T-cell-specific analyses, while remaining in all other analyses.

## Results

### Patient characteristics

Our overall cohort included 90 patients, of whom 70 (77.8%) were PWH and 20 (22.2%) were HIV-negative controls. The median age of the cohort was 58.7 years ± 9.5 years. Sixty-six patients (73.3%) were men, and 24 (26.7%) were women. Eight patients (8.9%) presented with AIDS-defining cancers, including non-Hodgkin lymphoma (*n* = 2) and cervical cancer (*n* = 6). The remaining 82 patients (91.1%) had non-AIDS-defining cancers, most commonly lung cancer (*n* = 35), hepatocellular cancer (HCC; *n* = 23), and gastric cancer (*n* = 9). Seventy-eight patients (86.7%) had stage III disease or higher. **Table 1** summarizes the demographic characteristics of the study participants. Following PSM, a matched cohort of 56 patients (37 in the HIV-positive group and 19 in the HIV-negative group) was included in the comparative analysis. As shown in **Table 2**, no significant differences in baseline characteristics were observed between the matched cohorts. In the HIV-positive group, 15 patients were coinfected with the hepatitis C virus (HCV) and five with the hepatitis B virus. In the HIV-negative group, one patient had concomitant HCV, and five had chronic hepatitis B (CHB).

**Table 1:** Demographics and baseline characteristics of the study cohort.

Variable	All patients (*N* = 90)	HIV-positive group (*n* = 70)	HIV-negative group (*n* = 20)	*p*-value
Age	59.11 ± 9.92	57.71 ± 9.58	64.00 ± 9.77	0.012
Sex				
Male	66	49	17	0.181
Female	24	21	3	
Tumor type				
Lung cancer	35	24	11	0.119
Liver cancer	23	17	6	
Gastric cancer	9	6	3	
AIDS-defined cancers	7	7	0	
Others	16	16	0	
Cancer stage				
< 3	12	8	4	0.282
≥ 3	78	62	16	
PD-1 inhibitors				
Tislelizumab	34	27	7	0.838
Camrelizumab	31	23	8	
Sintilimab	25	20	5	
Concurrent oncological regimens				
Combination targeted therapy	39	31	8	0.938
Combination chemotherapy	43	33	10	
Chemotherapy + targeted therapy	8	6	2	
Treatment line				
First	56	41	15	0.181
Second or above	34	29	5	
Cycles of PD-1 inhibitors	8 (2–63)	8 (2–63)	6 (2–34)	0.777
Baseline HIV viral load (copies/mL)				
Undetectable	48	48	–	–
> 40	9	9	–	–
Not available	13	13	–	–
Baseline CD4+ T-cell counts (cells/µL)				
< 100	4	4	–	–
100–200	15	15	–	–
≧ 200	50	50	–	–
Not available	1	1	–	–
Duration of ART (years)				
< 10	30	30	–	–
≧ 10	40	40	–	–

**Table 2:** Baseline characteristics compared between groups after propensity score matching.

Variable	Total (N = 56)	HIV-positive group (n = 37)	HIV-negative group (n = 19)	p-value
Age	61.9 ± 8.3	61.2 ± 7.8	63.1 ± 9.1	0.430
Sex				
Male	47	31	16	0.967
Female	9	6	3	
Tumor type				
Lung cancer	27	17	10	0.890
Liver cancer	19	13	6	
Gastric cancer	10	7	3	
Cancer stage				
I–III	33	21	12	0.645
IV	23	16	7	
PD-1 inhibitor				
Tislelizumab	19	13	6	0.919
Camrelizumab	24	16	8	
Sintilimab	13	8	5	
Treatment line				
First	40	26	14	0.789
Second or above	16	11	5	
Cycles	7.00	7.00	6.00	0.882

Among the 70 PWH enrolled, four had CD4^+^ T-cell counts below 100 cells/μL, and nine had detectable HIV RNA, ranging from 41 to 1.04 × 10^6^ copies/mL. All patients had received ART prior to initiating PD-1 inhibitors, with two cases having a treatment interval of less than 2 weeks. Thirty-eight patients received Biktarvy, 18 received Dovato, and six received tenofovir/lamivudine/efavirenz. Forty-one PWH had been on ART for more than 10 years.

### Treatment

Of the 90 cases, 35 patients (38.9%) received tislelizumab, 31 (34.4%) received camrelizumab, and 24 (26.7%) received sintilimab. In accordance with the inclusion and exclusion criteria, all included patients received PD-1 inhibitors in combination with targeted cancer therapy or chemotherapy. Before receiving anti-PD-1 antibodies, 34 patients (37.8%) had undergone frontline chemotherapy. Regarding concurrent regimens administered alongside PD-1 inhibitors, 43 patients received chemotherapy, 39 received targeted therapy, and eight received a combination of targeted therapy and chemotherapy. The median number of treatment cycles was seven (range: 2–63). The median follow-up duration for the matched cohort was 6.2 months (range: 2.6–14.2 months).

### Treatment response

Among the 37 PWH, six (16.2%) achieved a partial response, 13 (35.1%) had stable disease, and 18 (48.6%) experienced progressive disease, yielding an objective response rate (ORR) of 16.2% and a disease control rate (DCR) of 51.4%. In the HIV-negative cohort (*n* = 19), three patients achieved a Partial response (PR), six had SD, and 10 experienced PD, resulting in an ORR of 15.8% and a DCR of 47.4%. The median PFS was 11.5 months (95% CI: 6.8–16.2) for the HIV-positive group compared with 16.9 months (95% CI: NE–40.4) for the HIV-negative group. In the univariable Cox regression analysis, female sex (HR = 3.20, 95% CI: 1.25–8.18, *p* = 0.015), liver cancer (HR = 0.42, 95% CI: 0.19–0.95, *p* = 0.038), and chemotherapy alone (HR = 0.30, 95% CI: 0.13–0.71, *p* = 0.006) were significantly associated with PFS. However, in the multivariable model adjusting for baseline covariates, no factors reached independent statistical significance, though female sex (adjusted HR = 2.65, *p* = 0.069) and chemotherapy alone (adjusted HR = 0.29, *p* = 0.059) maintained distinct trends toward influencing survival outcomes. Multivariable Cox regression analysis showed that, after adjusting for confounders, HIV status was not independently associated with PFS (adjusted HR = 0.84, 95% CI: 0.37–1.91, *p* = 0.674) (**Table 3**).

**Table 3:** Univariable and multivariable Cox regression analysis of PFS in patients with cancer.

Variable	Univariable Cox regression			Multivariable Cox regression		
	HR	95% CI	*p*-value	HR	95% CI	*p*-value
Sex						
Male	1.00			1.00		
Female	3.20	1.25~8.18	0.015	2.65	0.93~7.56	0.069
Age	0.96	0.91~1.00	0.076	0.98	0.93~1.04	0.509
HIV status						
Positive	1.00					
Negative	0.84	0.37~1.91	0.674			
Tumor type						
Lung cancer	1.00			1.00		
Liver cancer	0.42	0.18~0.95	0.038	0.92	0.27~3.09	0.893
Gastric cancer	0.49	0.16~1.49	0.208	1.25	0.24~6.56	0.790
Cancer stage						
I–III	1.00					
IV	0.66	0.30~1.45	0.301			
PD-1 inhibitor						
Tislelizumab	1.00					
Camrelizumab	1.71	0.69~4.22	0.247			
Sintilimab	2.17	0.81~5.85	0.125			
Treatment line						
First	1.00					
Second or above	1.06	0.48~2.36	0.887			
Concurrent oncological regimens						
Targeted therapy	1.00			1.00		
Chemotherapy	0.30	0.13~0.71	0.006	0.29	0.08~1.05	0.059
Targeted therapy + chemotherapy	0.78	0.18~3.44	0.746	0.71	0.12~4.33	0.715

**Figure 2:**
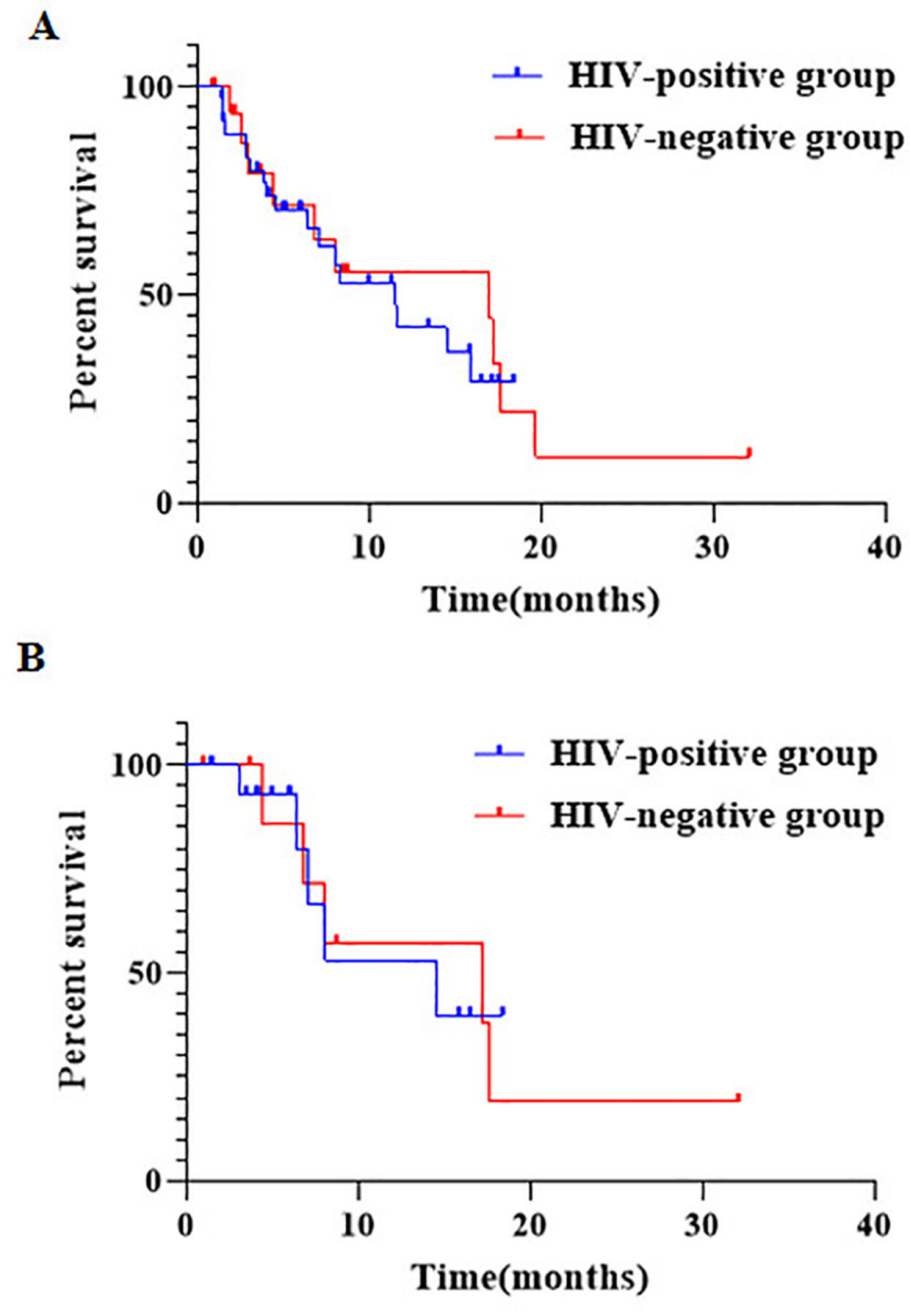
Kaplan–Meier curves of progression-free survival (PFS) in the propensity score-matched groups. **(A)** PFS comparison between the HIV-positive and HIV-negative groups. **(B)** PFS comparison between the HIV-positive and HIV-negative patients in the lung cancer subgroup.

We also conducted a subgroup analysis of patients with lung cancer (*N* = 27), which included 17 patients in the HIV-positive group and 10 in the HIV-negative group. In the HIV-positive cohort, four patients (23.5%) achieved a PR, eight (47.0%) had SD, and five (29.4%) experienced PD, resulting in an ORR of 23.5% and a DCR of 70.6%. In the HIV-negative cohort, three patients (30.0%) achieved a PR, two (20.0%) had SD, and five (50.0%) experienced PD, resulting in an ORR of 30.0% and a DCR of 50.0%. The median PFS was 14.5 months (95% CI: 4.9–24.2) for the HIV-positive group and 17.2 months (95% CI: NE–36.0) for the HIV-negative group, with no significant difference observed in treatment response between the two cohorts. The corresponding PFS curves are shown in **Figure 2**.

Among four HIV-positive patients with advanced immunosuppression (CD4+ T-cell counts below 100 cells/μL), none achieved PR, one had SD, and three experienced PD (ORR = 0.0%; DCR = 25.0%). Among the nine HIV-positive patients with detectable HIV RNA, none achieved PR, four had SD, and five experienced PD (ORR = 0.0%; CDR = 44.4%). The clinical characteristics, treatment responses, and toxicities of these individuals are summarized in **Table 4**.

**Table 4:** Clinical characteristics, therapeutic response, and toxicities of the 11 patients presenting with detectable baseline HIV RNA (*n* = 9) and/or advanced immunosuppression (*n* = 4).

Patient	Sex	Age (years)	Cancer and stage	PD-1 inhibitor	HIV RNA at baseline (copies/mL)	HIV RNA after PD-1 inhibitors (copies/mL)	CD4+ T-cell count at baseline (cells/μL)	CD4+ T-cell count at 24 weeks (cells/μL)	Response	Toxicity
1	Male	35	Lung cancer III	Tislelizumab	13,932	ND	122	179	PD	NO
2	Male	69	Lung cancer III	Sintilimab	1.62 × 105	ND	210	219	SD	NO
3	Male	69	Lung cancer III	Sintilimab	6,754	ND	318	402	SD	Hypothyroidism
4	Male	51	Liver cancer III	Sintilimab	1,323	ND	165	258	PD	Hypothyroidism
5^a^	Male	78	Lung cancer IV	Camrelizumab	1.04 × 106	NA	36	248	SD	NO
6	Male	55	Esophageal cancer III	Camrelizumab	59	ND	321	345	PD	Hypothyroidism
7	Male	48	Esophageal cancer IV	Camrelizumab	41	NA	399	400	SD	NO
8^a^	Male	55	Esophageal cancer IV	Tislelizumab	542	54	69	154	PD	NO
9	Male	27	Gastric cardia adenocarcinoma IV	Sintilimab	3,049	44	208	282	PD	NO
10	Male	63	Lung cancer III	Tislelizumab	ND	ND	49	135	PD	NO
11	Male	69	Nasopharyngeal carcinoma III	Camrelizumab	ND	ND	73	137	PD	NO

a Patients 5 and 8 had detectable HIV RNA and concurrent CD4+ T-cell counts < 100 cells/μL at baseline.

*NA*, not available; *ND*, not detected; *NO*, not observed.

### Safety outcomes

Among the matched cohort (*N* = 56), treatment-related adverse events (TRAEs) considered potentially attributable to PD-1 inhibitors were observed in 18 patients (32.1%), 11 in the HIV-positive group and seven in the HIV-negative group. The majority of these events were immune-related adverse reactions. Most TRAEs were low-grade (*n* = 15 for grade 1 or 2), whereas severe TRAEs (grade ≥ 3) affected the remaining three patients, all within the HIV-positive group. In the latter cohort, hypothyroidism was the most frequent TRAE, occurring in five patients. One patient experienced pneumonitis that necessitated the permanent suspension of PD-1 inhibitor therapy. Another patient developed severe oral mucositis after 15 cycles of camrelizumab, presenting with trismus (restricted mouth opening) and a 10-kg weight loss. Notably, no cases of immune reconstitution inflammatory syndrome were observed, even among the two patients who had recently initiated ART.

Conversely, documented TRAEs in the HIV-negative group included hypothyroidism (*n* = 4), whereas hepatic injury, rash, and reactive cutaneous capillary endothelial proliferation (RCCEP) each occurred in a single patient. Overall, four patients permanently discontinued immunotherapy due to TRAEs—specifically immune-related pneumonitis, hypophysitis, severe oral mucositis, and liver injury—all of whom belonged to the HIV-positive group. No treatment-related mortality was recorded in either group. The complete distribution and grading of TRAEs are presented in **Table 5**.

**Table 5:** Treatment-related adverse events possibly attributed to PD-1 inhibitors.

TRAES	HIV-positive group (*n* = 37)	HIV-negative group (*n* = 19)	Grade ≥ 3 TRAEs (All HIV^+^)
Thyroiditis	5	4	
Hepatic injury	2	1	1
Hypophysitis	2	0	
Pneumonia	1	0	1
Gastrointestinal mucositis	1	0	
Allergic-like reaction	0	1	
Systemic ulceration	1	0	1
Rash	1	1	
RCCEP	1	1	

Data are presented as raw counts (*n*). Individual patients may experience two or more distinct TRAEs.*RCCEP*, reactive cutaneous capillary endothelial proliferation; *TRAEs*, treatment-related adverse events.

### CD4^+^ T-cell and HIV monitoring

Longitudinal CD4^+^ T-cell counts were monitored in 69 patients within the HIV-positive group. At the end of treatment, the median CD4^+^ T-cell count was 283 cells/μL (range: 18–918 cells/μL). Among these individuals, 22 patients exhibited an elevation in CD4^+^ T-cell counts, 34 showed a decline, and 13 remained stable. Notably, all four patients who presented with baseline CD4^+^ T-cell counts < 100 cells/μL exhibited an increase following therapy.

Regarding virological control, baseline plasma HIV RNA data were available for 57 individuals, with 48 cases below the detection threshold. Among these 48 patients, 16 consistently maintained undetectable viral loads throughout the entire duration of PD-1 inhibitor therapy. Of the nine patients with initially detectable HIV viral loads (range: 41 to 1.04 × 10^6^ copies/mL), five achieved complete viral suppression and two exhibited significant reductions, while two lacked posttreatment data. No instances of viral rebound were observed during immunotherapy, and all patients demonstrated full adherence to ART.

## Discussion

The intersection of HIV/AIDS and concurrent malignancies presents a profound public health challenge. Driven by increased population longevity, widespread coinfection with oncogenic viruses, lifestyle factors such as tobacco use, and the cumulative genotoxic or metabolic effects of long-term antiretroviral regimens, the risk of developing non-AIDS-defining malignant tumors among PWH has risen substantially (28, 29). Several trials have documented cancer treatment outcomes in PWH (30–32); however, HIV-specific oncological guidelines remain scarce. Previous studies have demonstrated that ICIs, such as pembrolizumab or durvalumab, are well-tolerated in PWH with CD4^+^ T-cell counts greater than 100 cells/μL; nevertheless, further investigations are warranted to fully characterize the safety and efficacy of novel immunotherapy regimens in this population (33, 34).

In this retrospective, observational study, we report the clinical characteristics, treatment response, and tolerability of PD-1 inhibitors in patients with cancer, comparing those with and without HIV, including individuals with advanced immunosuppression (CD4^+^ T-cell count < 100 cells/μL) or uncontrolled HIV RNA. Most major clinical trials explicitly exclude individuals with uncontrolled viremia or profound T-cell depletion as a precaution. This strict selection bias creates a substantial knowledge gap, as clinicians frequently encounter PWH who do not meet these idealized trial criteria but still require effective oncology treatment. Our study directly addresses this gap by evaluating a more representative real-world cohort.

In our overall cohort of 90 cancer patients (70 HIV-positive and 20 HIV-negative subjects), men represented 73.3% of the sample, and lung cancer was the predominant malignancy, which is in line with the epidemiological data on AIDS and cancer in China (35, 36). The PD-1 inhibitors included in this study were all developed and manufactured by Chinese companies and have been included in China’s National Reimbursement Drug List, making them more affordable and accessible to the Chinese population (37).

In the present study, no statistically significant differences in treatment response were observed between the HIV-positive and HIV-negative cohorts after PSM. This equivalence was also observed within the lung cancer subgroup, aligning closely with previous findings (38, 39). Wu et al. reported an ORR of 33.3% and a DCR of 62.5% for tislelizumab in PWH with bladder cancer (38). Another study reported that among 20 HIV-positive patients treated with durvalumab, the ORR was 25%, and the DCR was 56% (33). In contrast, a phase 1 trial involving 30 participants with HIV and advanced cancer reported that only three patients achieved an objective response after receiving pembrolizumab (40). These disparate outcomes may be attributed not only to small sample sizes, heterogeneous follow-up durations, and variations in primary tumor types but also to distinct biochemical and pharmacodynamic properties among the antibodies evaluated, specifically regarding target affinity and Fc-receptor engineering across anti-PD-1 and anti-PD-L1 agents.

Notably, a recent systematic scoping review pooling data from 107 cases across 19 studies demonstrated an encouraging macrolevel efficacy profile, reporting that 27.1% of PWH achieved a PR and 23.4% had SD in response to PD-1 inhibitors (24). Another systematic review evaluating checkpoint inhibition across 762 PWH with non-small cell lung cancer demonstrated that the ORR varied from 13% to 75%, the DCR ranged from 47% to 62.5%, and the median PFS ranged from 3.0 to 6.3 months (41). Taken together, these data strongly indicate that HIV status is not associated with a diminution in the clinical efficacy of PD-1 inhibitors. These accumulated findings further support the emerging consensus that immune checkpoint inhibitors demonstrate encouraging antitumor activity in PWH presenting with concurrent malignancies, although larger prospective trials remain limited.

Although macrolevel evidence points to general therapeutic parity, variations emerge when evaluating patients with severe immune depletion. For instance, Xiong et al. reported on a small subset of two patients with CD4^+^ T-cell counts < 100 cells/μL, both of whom demonstrated favorable objective responses to immunotherapy, suggesting no clear association between baseline CD4^+^ T-cell counts and therapeutic benefit (42). Conversely, within our subgroup of patients presenting with advanced immunosuppression (CD4^+^ T-cell count < 100 cells/μL) and uncontrolled HIV RNA copy numbers, no patients achieved a PR. This discrepancy underscores the need for clinical caution; the true therapeutic impact within this deeply vulnerable subgroup remains to be fully elucidated by larger-scale studies.

Regarding longitudinal immune dynamics, posttreatment changes in CD4^+^ T-cell counts within our cohort demonstrated a highly reassuring trend of immunologic stabilization and recovery. Strikingly, within our most vulnerable subgroup—individuals presenting with baseline advanced immunosuppression (CD4^+^ count < 100 cells/mL)—every patient experienced a robust and clinically meaningful increase in T-cell counts by week 24, exemplified by one patient whose count increased from a baseline of 36 to 248 cells/mL. Across the broader cohort, trajectories remained highly stable or upward trending, with no instances of severe immunological decline. These observations further validate established real-world data indicating that immune checkpoint blockade does not exert a deleterious effect on overall T-cell homeostasis or drive virological failure in PWH (43, 44). Crucially, this immune reconstitution occurred without any detectable reactivation of the HIV viral load, providing definitive real-world evidence that PD-1 inhibitors do not induce viral replication or compromise virological control. Furthermore, the robust CD4^+^ T-cell recoveries observed among our severely immunosuppressed patients suggest that, under strict virological monitoring, therapeutic immune reconstitution is entirely achievable even within subsets traditionally excluded from landmark trials due to safety concerns.

Clinical studies have further demonstrated that immunotherapy is safe and well-tolerated in PWH with cancer. In this study, the overall incidence of treatment-related adverse events attributable to PD-1 inhibition was 32.1%, which is nominally higher than the 20.0% any-grade immune-related adverse event (irAE) rate reported by Gonzalez-Cao et al. in their multicenter prospective study (33)—a safety benchmark subsequently validated on a larger scale by El Zarif et al. in the 2023 CATCH-IT Consortium cohort of 390 patients (39).

Within our matched populations, the patient who developed rapid weight loss demonstrated symptomatic improvement and a partial recovery of body weight following the timely discontinuation of camrelizumab. This case underscores the need for clinicians to promptly manage adverse reactions to minimize treatment-associated morbidity. Crucially, treatment-limiting toxicities in our study were rare; severe (grades 3–4) TRAEs were observed in only 5.4% of cases (three of 56 patients) across the total matched cohort. Although these severe events, alongside the four subsequent treatment discontinuations, were confined to the HIV-positive group, this frequency aligns well with the expected thresholds reported in the broader oncology population (25). These findings reinforce the growing consensus that immune checkpoint inhibitors maintain a favorable safety and tolerability profile in PWH under appropriate monitoring.

The immune profile of individuals with chronic HIV infection closely resembles the immunosuppressive microenvironment observed in oncology, as both states are characterized by T-cell exhaustion and immune evasion (45). Blocking the PD-1/PD-L1 signaling pathway can reverse the exhaustion of HIV-specific CD8^+^ T cells, restore cytokine production and cellular proliferation, and concurrently enhance HIV transcription and RNA translation. This process facilitates viral elimination through virus-induced apoptosis or immune-mediated clearance, thereby reducing the HIV reservoir (46). It has been documented that an HIV-infected patient with concurrent non-small cell lung cancer exhibited a significant reduction in the HIV reservoir following nivolumab administration (47). Conversely, in an HIV-infected patient with melanoma, no sustained depletion of reservoir CD4^+^ T cells was observed after pembrolizumab therapy (48). In our study, HIV viral load was evaluable in five patients throughout the treatment course. However, due to its retrospective design, our study lacked access to HIV reservoir data, precluding an evaluation of this potential therapeutic benefit. Consequently, prospective studies are needed to further elucidate the precise role of the PD-1 signaling pathway in modulating the HIV reservoir.

Compared with prior clinical trials, our study featured a larger sample size and more lenient inclusion criteria to assess the real-world safety and efficacy of PD-1 inhibitors in PWH, thereby aligning more closely with clinical reality. Nevertheless, several limitations must be acknowledged. First, as a single-center retrospective study, selection bias is unavoidable. Second, only four patients presented with baseline CD4^+^ T-cell counts below 100 cells/μL; this sample size is insufficient to draw robust conclusions regarding treatment efficacy in severely immunosuppressed individuals. Third, because PD-1 inhibitor monotherapy was a predefined exclusion criterion for this study, three patients receiving this modality were excluded from the analysis; therefore, our findings reflect the outcomes of combination regimens exclusively and cannot be generalized to monotherapy settings. Fourth, the lack of longitudinal HIV reservoir data precludes an assessment of the potential role of checkpoint inhibition in modulating viral latency. Fifth, adverse events were collected primarily from medical records, which may have introduced underreporting or overlooked transient adverse reactions. Consequently, large-scale, prospective studies, ideally with multicenter designs, are warranted to further validate our findings and clarify these relationships.

## Conclusions

In conclusion, our study demonstrates that PD-1 inhibitors exhibit promising efficacy and manageable safety profiles in PWH with concurrent malignancies. Our findings suggest that HIV status itself does not adversely affect the treatment outcomes of PD-1 inhibitor therapy. Nevertheless, whether a low CD4^+^ T-cell count or a detectable HIV RNA load influences these outcomes remains to be definitively determined. Critically, the observation that severe (grade ≥ 3) toxicities and subsequent treatment discontinuations were confined to the HIV-positive cohort highlights the need for vigilant clinical monitoring in this patient population.

## Data Availability

The datasets presented in this study can be found in online repositories. The names of the repository/repositories and accession number(s) can be found in the article/supplementary material.
