# Supplementary figures and images for: Real-world efficacy and safety of PD-1 inhibitors in patients living with HIV and cancer: a retrospective cohort study
Source: Front Oncol. 2026 Jun 24;16:1846602. doi: 10.3389/fonc.2026.1846602 (PMC13341521; doi:10.3389/fonc.2026.1846602)

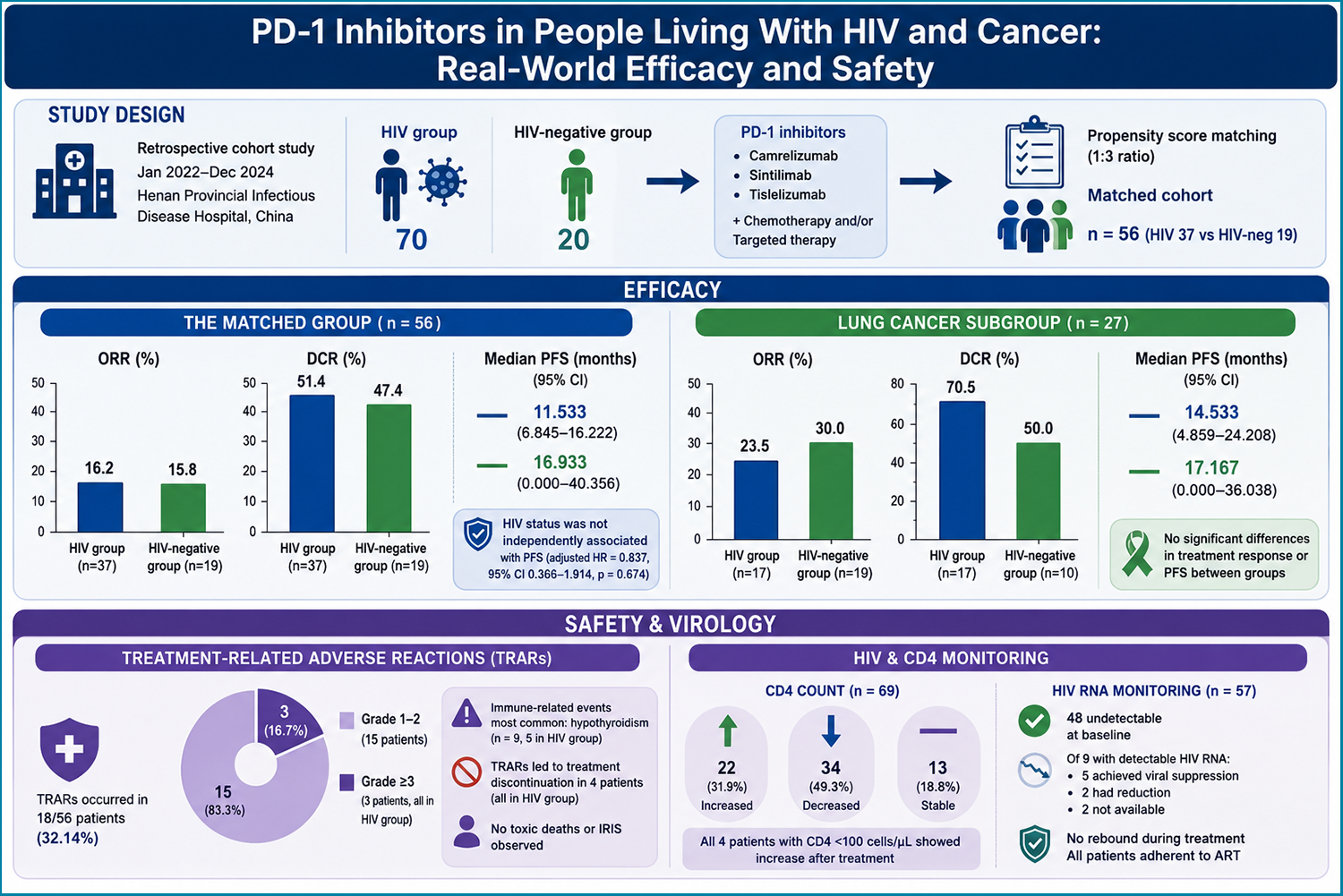

Supplement: Supplementary file 1 [file Image1.png]
